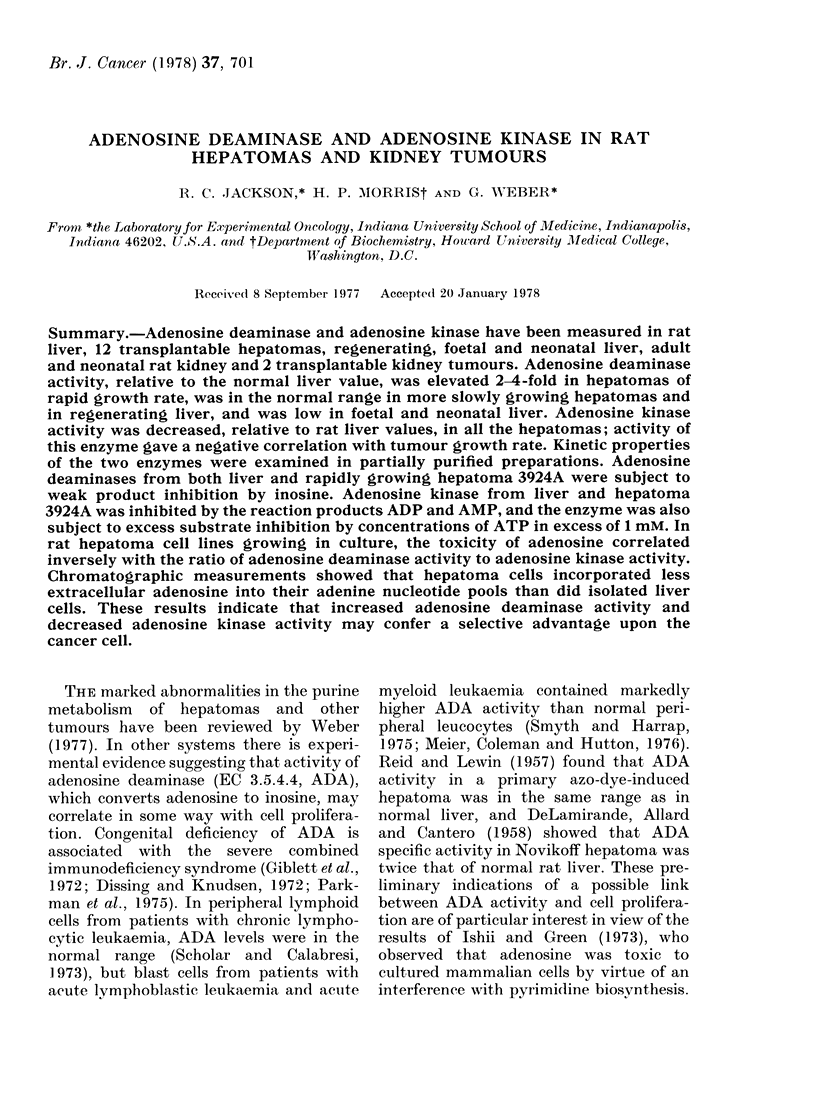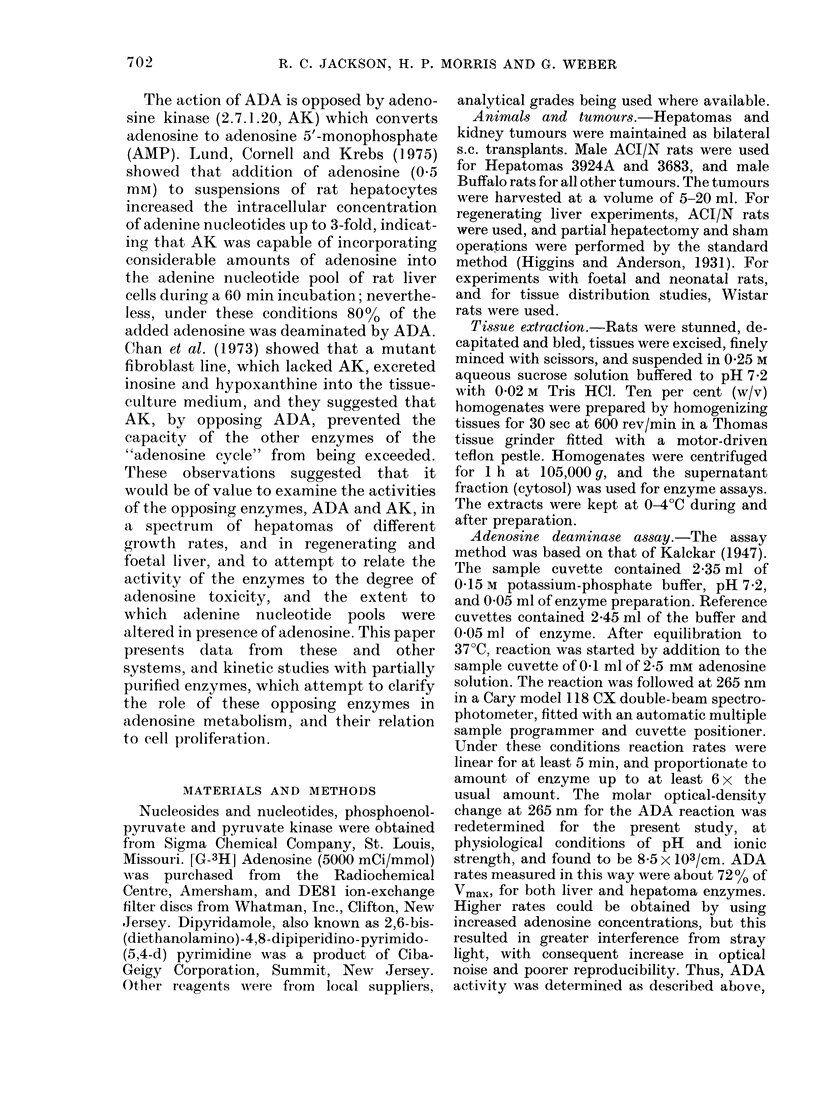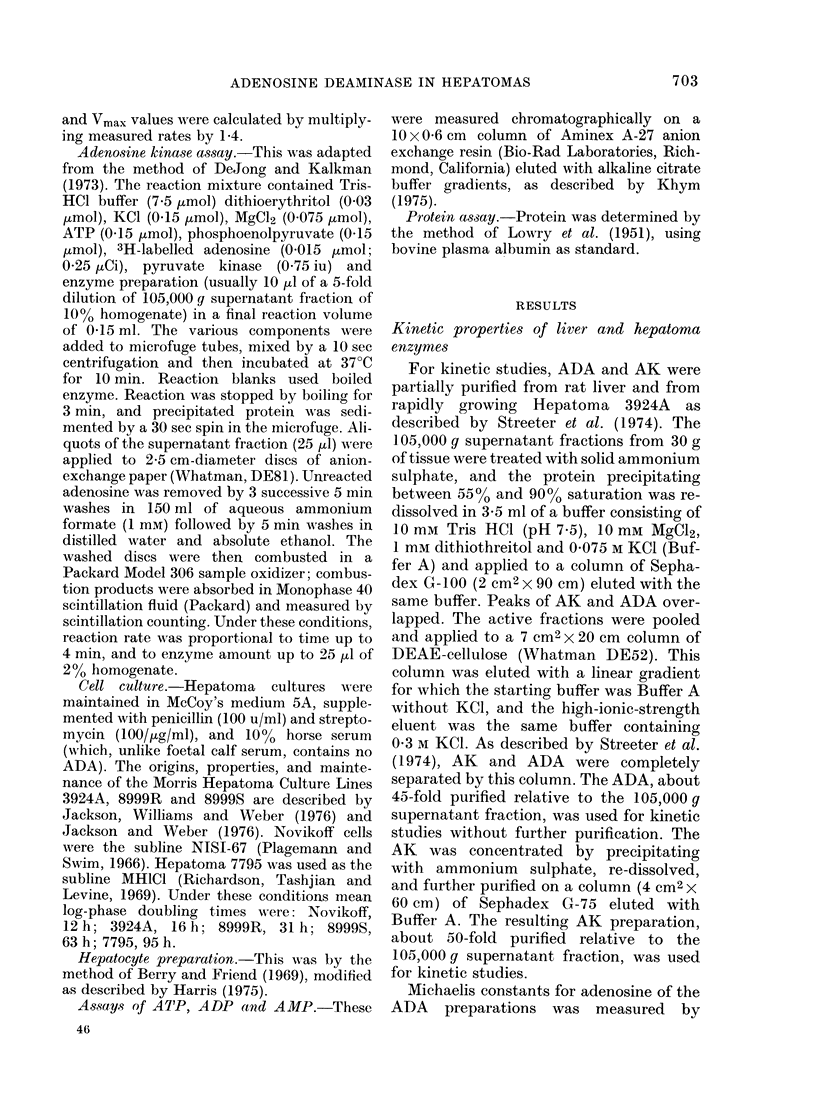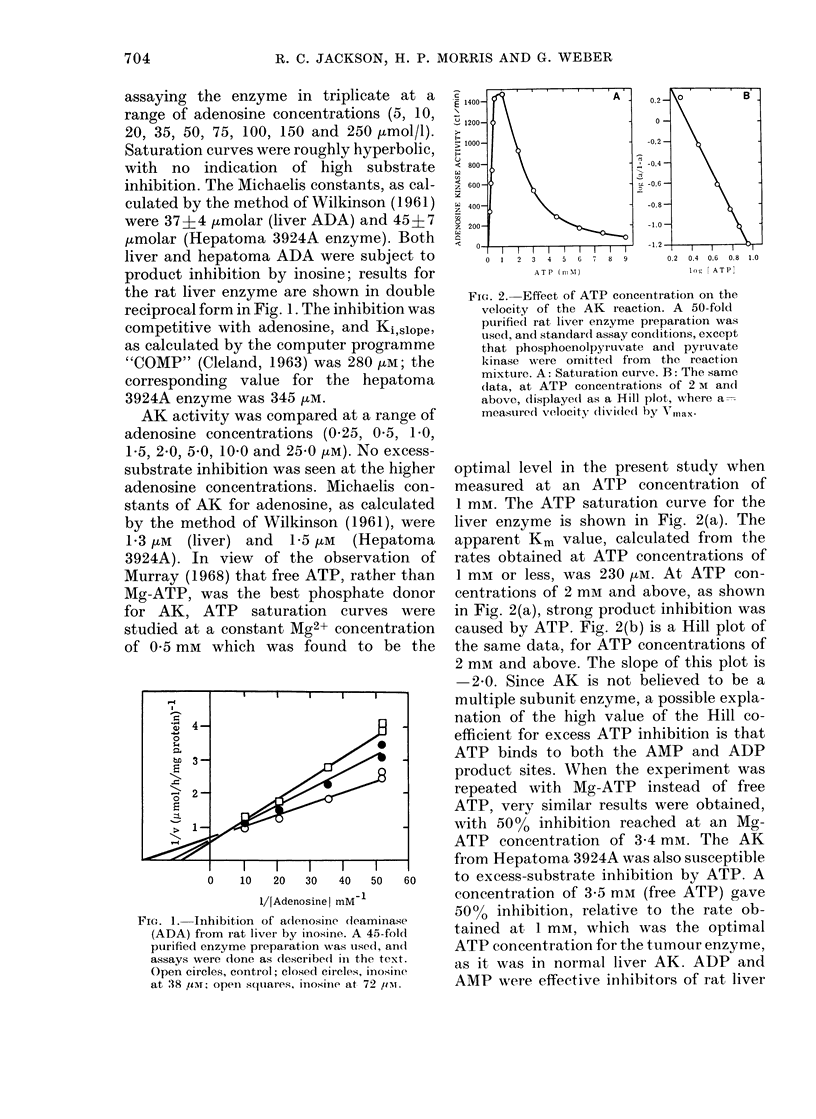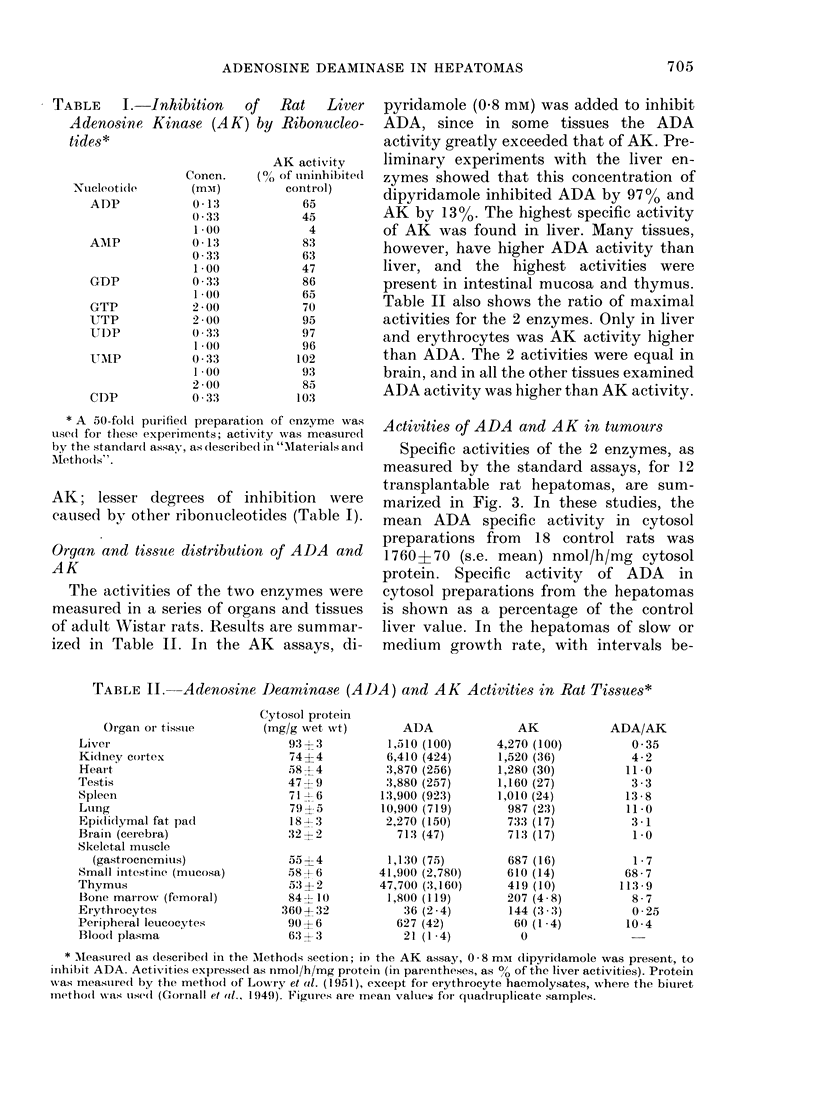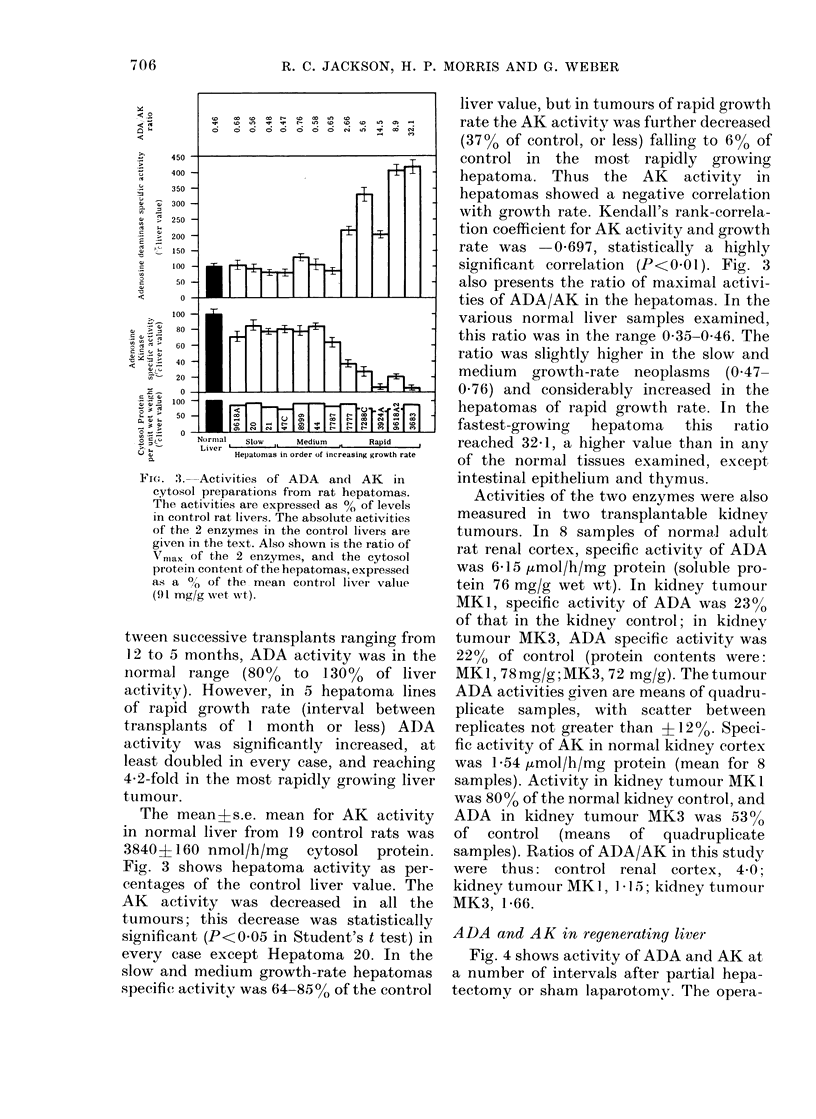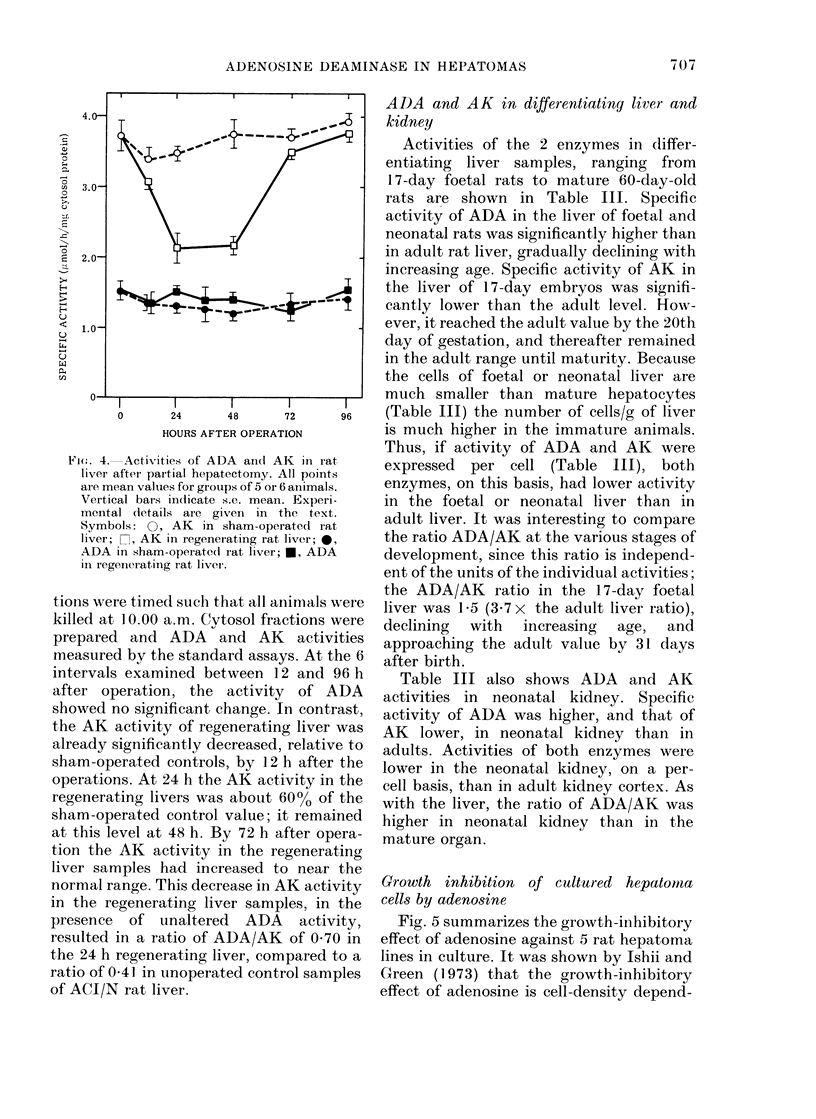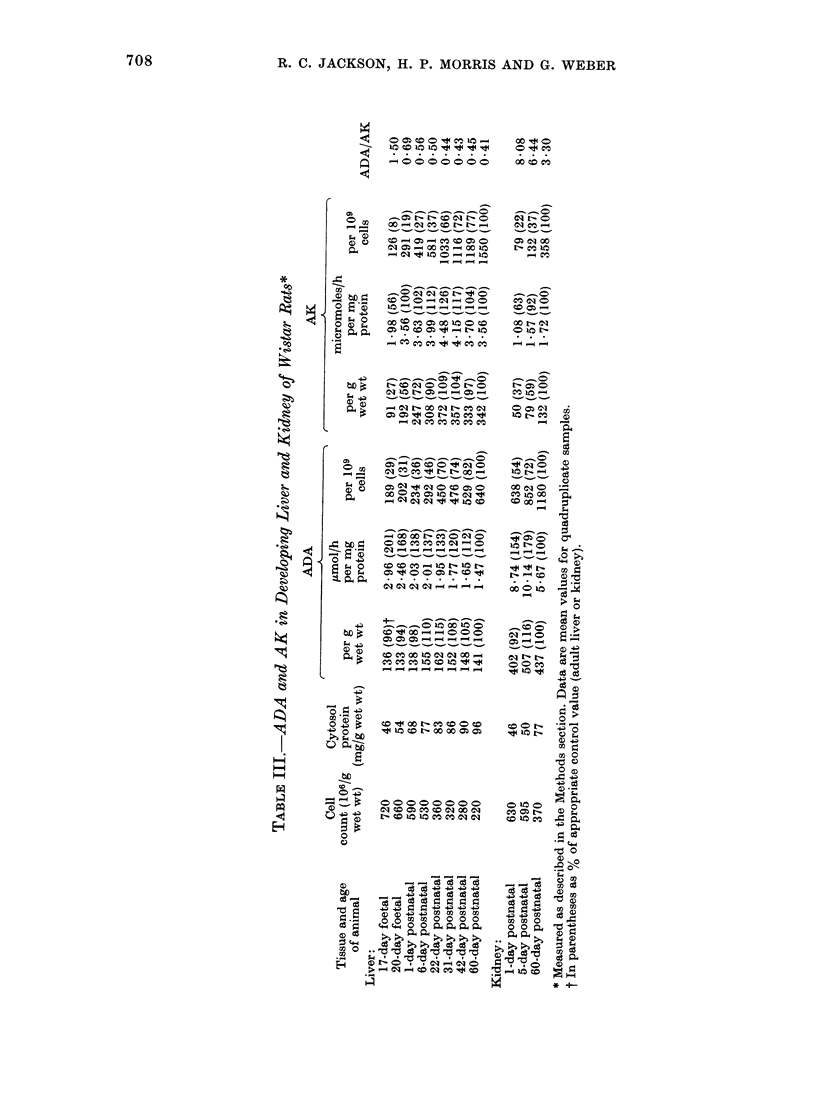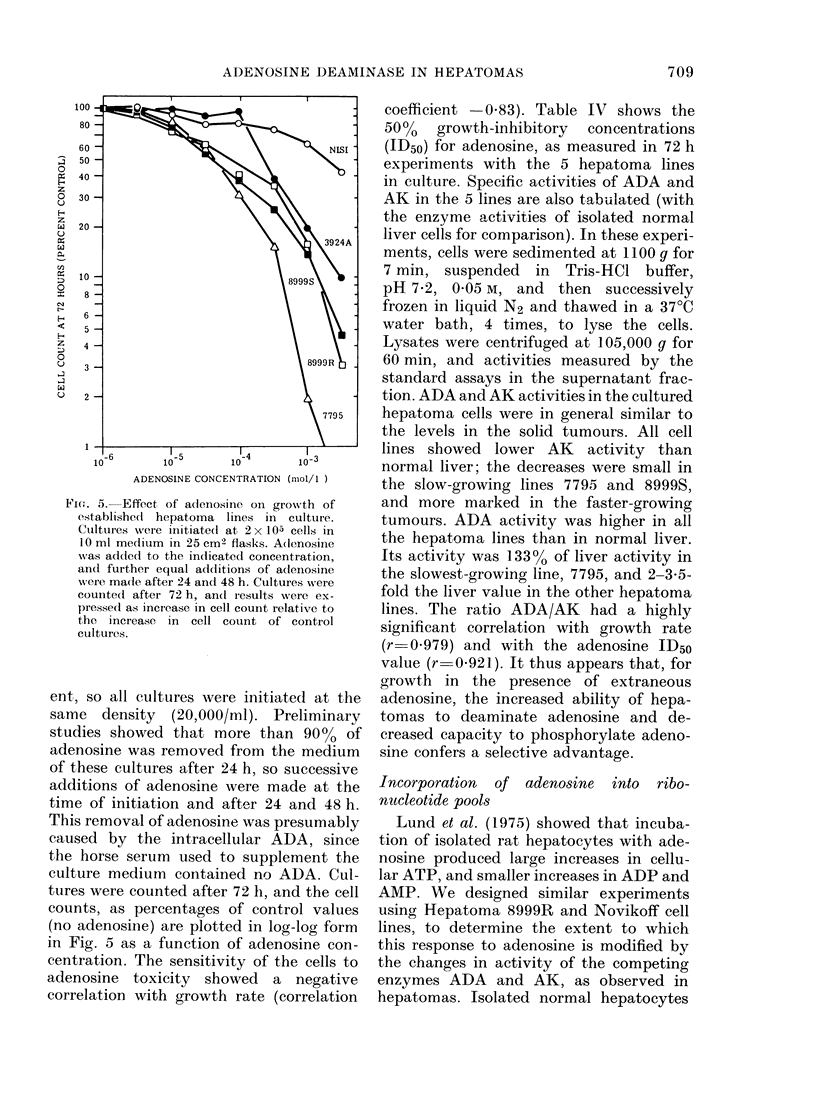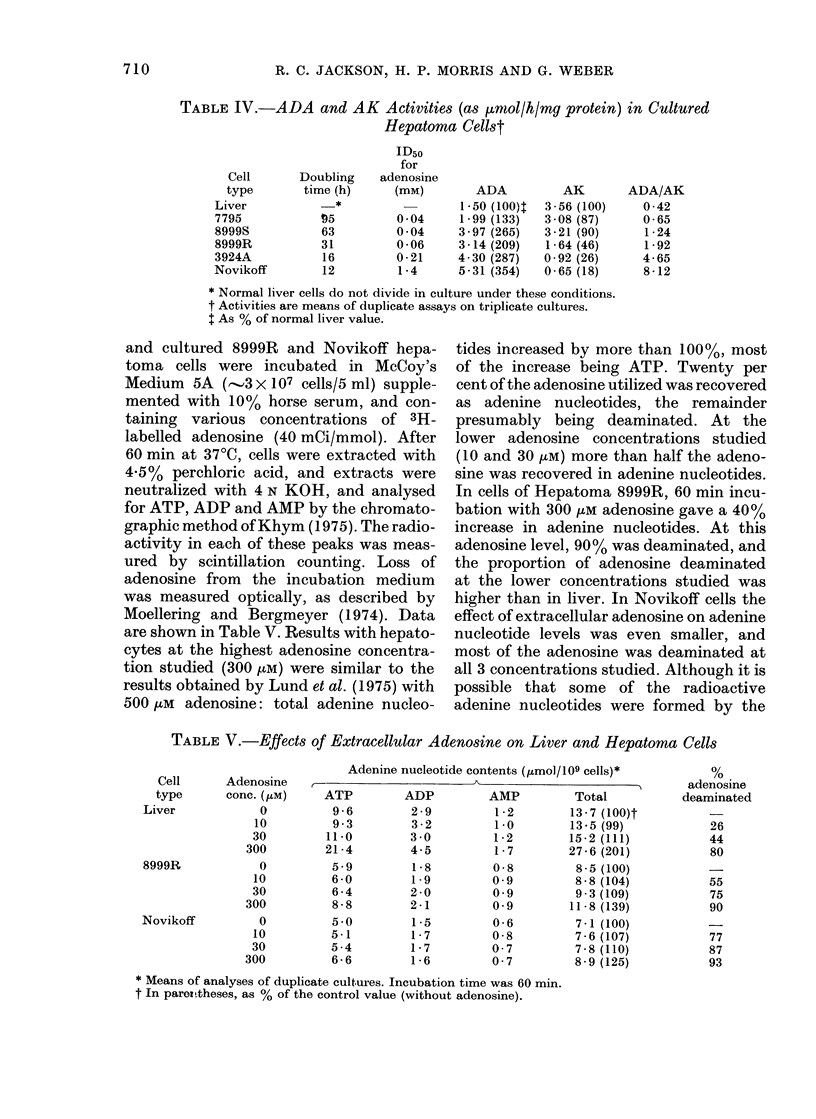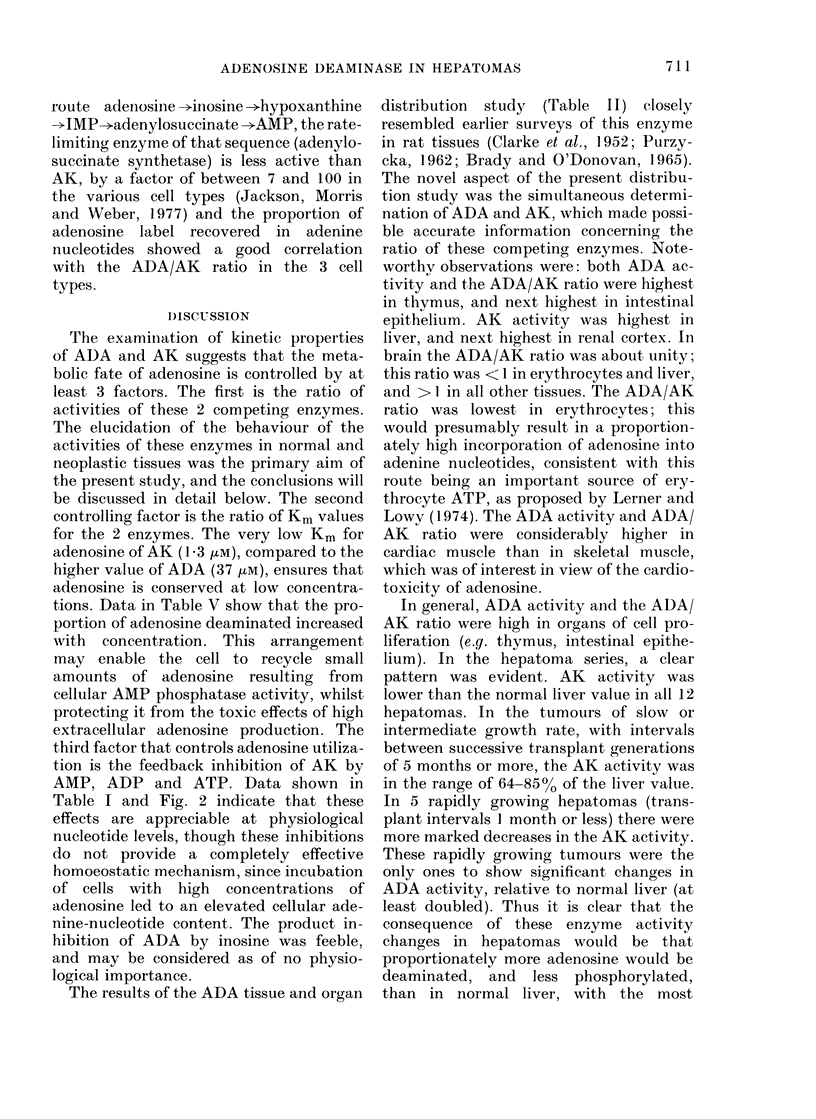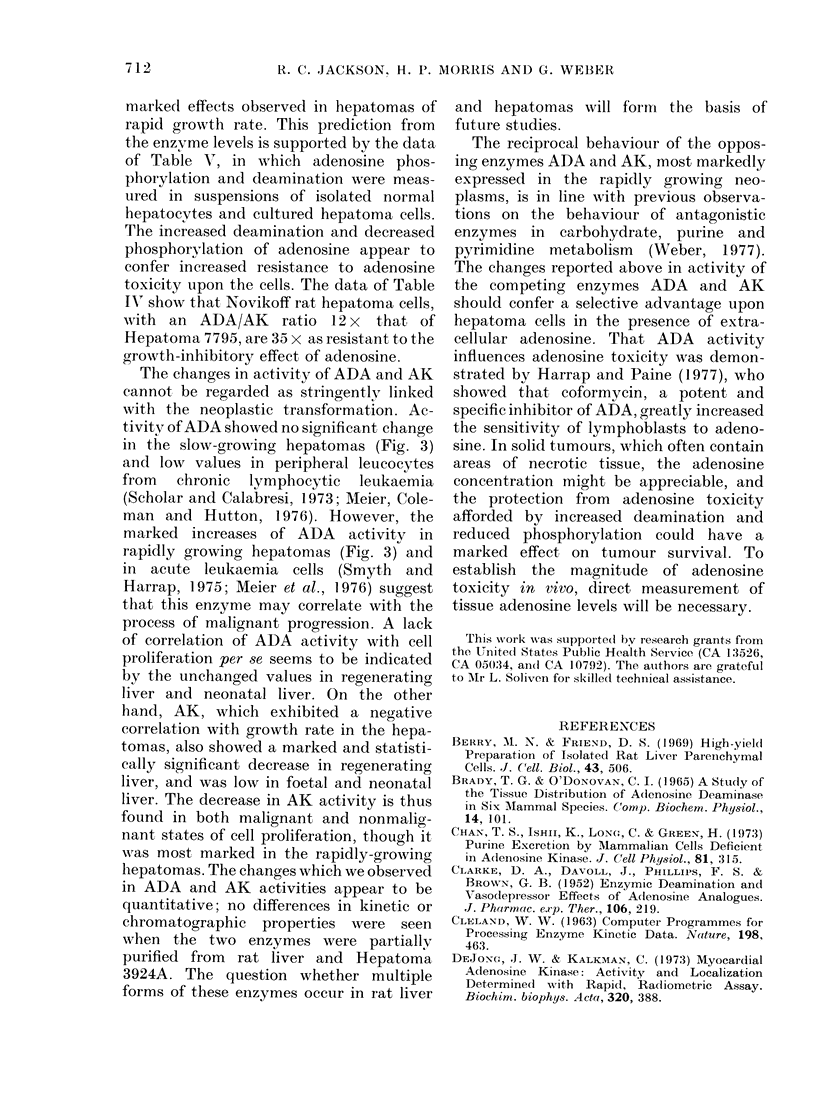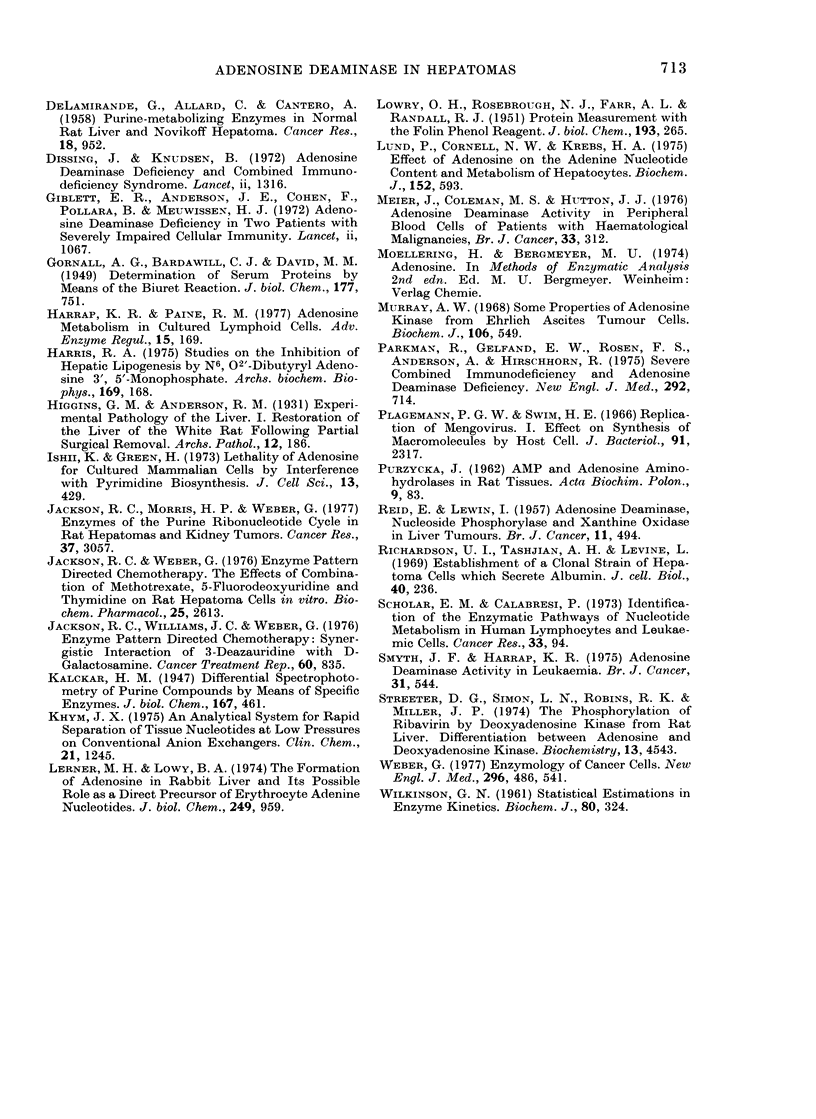# Adenosine deaminase and adenosine kinase in rat hepatomas and kidney tumours.

**DOI:** 10.1038/bjc.1978.107

**Published:** 1978-05

**Authors:** R. C. Jackson, H. P. Morris, G. Weber

## Abstract

Adenosine deaminase and adenosine kinase have been measured in rat liver, 12 transplantable hepatomas, regenerating, foetal and neonatal liver, adult and neonatal rat kidney and 2 transplantable kidney tumours. Adenosine, deaminase activity, relative to the normal liver value, was elevated 2-4 fold in hepatomas of rapid growth rate, was in the normal range in more slowly growing hepatomas and in regernerating liver, and was low in foetal and neonatal liver. Adenosine kinase activity was decreased, relative to rat liver values, in all the hepatomas; activity of this enzyme gave a negative correlation with tumour growth rate. Kinetic properties of the two enzymes were examined in partially purified preparations. Adenosine deaminases from both liver and rapidly growing hepatoma 3924A were subject to weak product inhibition by inosine. Adenosine kinase from liver and hepatoma 3924A was inhibited by the reaction products ADP and AMP, and the enzyme was also subject to excess substrate inhibition by concentrations of ATP in excess of 1 mM. In rat hepatoma cell lines growing in culture, the toxicity of adenosine correlated inversely with the ratio of adenosine deaminase activity to adenosine kinase activity. Chromatographic measurements showed that hepatoma cells incorporated less extracellular adenosine into their adenine nucleotide pools than did isolated liver cells. These results indicate that increased adenosine deaminase activity and decreased adenosine kinase activity may confer a selective advantage upon the cancer cell.


*Br. J. Cancer* (1978) **37**, 701

### ADENOSINE DEAMINASE AND ADENOSINE KINASE IN RAT HEPATOMAS AND KIDNEY TUMOURS

R. C. JACKSON,* H. P. MORRIS† AND G. WEBER*

*From *the Laboratory for Experimental Oncology, Indiana University School of Medicine, Indianapolis, Indiana 46202, U.S.A. and †Department of Biochemistry, Howard University Medical College, Washington, D.C.*

Received 8 September 1977 Accepted 20 January 1978

**Summary.—Adenosine deaminase and adenosine kinase have been measured in rat liver, 12 transplantable hepatomas, regenerating, foetal and neonatal liver, adult and neonatal rat kidney and 2 transplantable kidney tumours. Adenosine deaminase activity, relative to the normal liver value, was elevated 2–4-fold in hepatomas of rapid growth rate, was in the normal range in more slowly growing hepatomas and in regenerating liver, and was low in foetal and neonatal liver. Adenosine kinase activity was decreased, relative to rat liver values, in all the hepatomas; activity of this enzyme gave a negative correlation with tumour growth rate. Kinetic properties of the two enzymes were examined in partially purified preparations. Adenosine deaminases from both liver and rapidly growing hepatoma 3924A were subject to weak product inhibition by inosine. Adenosine kinase from liver and hepatoma 3924A was inhibited by the reaction products ADP and AMP, and the enzyme was also subject to excess substrate inhibition by concentrations of ATP in excess of 1 mM. In rat hepatoma cell lines growing in culture, the toxicity of adenosine correlated inversely with the ratio of adenosine deaminase activity to adenosine kinase activity. Chromatographic measurements showed that hepatoma cells incorporated less extracellular adenosine into their adenine nucleotide pools than did isolated liver cells. These results indicate that increased adenosine deaminase activity and decreased adenosine kinase activity may confer a selective advantage upon the cancer cell.**

THE marked abnormalities in the purine metabolism of hepatomas and other tumours have been reviewed by Weber (1977). In other systems there is experimental evidence suggesting that activity of adenosine deaminase (EC 3.5.4.4, ADA), which converts adenosine to inosine, may correlate in some way with cell proliferation. Congenital deficiency of ADA is associated with the severe combined immunodeficiency syndrome (Giblett *et al.*, 1972; Dissing and Knudsen, 1972; Parkman *et al.*, 1975). In peripheral lymphoid cells from patients with chronic lymphocytic leukaemia, ADA levels were in the normal range (Scholar and Calabresi, 1973), but blast cells from patients with acute lymphoblastic leukaemia and acute myeloid leukaemia contained markedly higher ADA activity than normal peripheral leucocytes (Smyth and Harrap, 1975; Meier, Coleman and Hutton, 1976). Reid and Lewin (1957) found that ADA activity in a primary azo-dye-induced hepatoma was in the same range as in normal liver, and DeLamirande, Allard and Cantero (1958) showed that ADA specific activity in Novikoff hepatoma was twice that of normal rat liver. These preliminary indications of a possible link between ADA activity and cell proliferation are of particular interest in view of the results of Ishii and Green (1973), who observed that adenosine was toxic to cultured mammalian cells by virtue of an interference with pyrimidine biosynthesis.

The action of ADA is opposed by adenosine kinase (2.7.1.20, AK) which converts adenosine to adenosine 5′-monophosphate (AMP). Lund, Cornell and Krebs (1975) showed that addition of adenosine (0·5 mM) to suspensions of rat hepatocytes increased the intracellular concentration of adenine nucleotides up to 3-fold, indicating that AK was capable of incorporating considerable amounts of adenosine into the adenine nucleotide pool of rat liver cells during a 60 min incubation; nevertheless, under these conditions 80% of the added adenosine was deaminated by ADA. Chan *et al.* (1973) showed that a mutant fibroblast line, which lacked AK, excreted inosine and hypoxanthine into the tissue-culture medium, and they suggested that AK, by opposing ADA, prevented the capacity of the other enzymes of the "adenosine cycle" from being exceeded. These observations suggested that it would be of value to examine the activities of the opposing enzymes, ADA and AK, in a spectrum of hepatomas of different growth rates, and in regenerating and foetal liver, and to attempt to relate the activity of the enzymes to the degree of adenosine toxicity, and the extent to which adenine nucleotide pools were altered in presence of adenosine. This paper presents data from these and other systems, and kinetic studies with partially purified enzymes, which attempt to clarify the role of these opposing enzymes in adenosine metabolism, and their relation to cell proliferation.

### MATERIALS AND METHODS

Nucleosides and nucleotides, phosphoenolpyruvate and pyruvate kinase were obtained from Sigma Chemical Company, St. Louis, Missouri. [G-$^3$H] Adenosine (5000 mCi/mmol) was purchased from the Radiochemical Centre, Amersham, and DE81 ion-exchange filter discs from Whatman, Inc., Clifton, New Jersey. Dipyridamole, also known as 2,6-bis-(diethanolamino)-4,8-dipiperidino-pyrimido-(5,4-d) pyrimidine was a product of Ciba-Geigy Corporation, Summit, New Jersey. Other reagents were from local suppliers, analytical grades being used where available.

*Animals and tumours.*—Hepatomas and kidney tumours were maintained as bilateral s.c. transplants. Male ACI/N rats were used for Hepatomas 3924A and 3683, and male Buffalo rats for all other tumours. The tumours were harvested at a volume of 5–20 ml. For regenerating liver experiments, ACI/N rats were used, and partial hepatectomy and sham operations were performed by the standard method (Higgins and Anderson, 1931). For experiments with foetal and neonatal rats, and for tissue distribution studies, Wistar rats were used.

*Tissue extraction.*—Rats were stunned, decapitated and bled, tissues were excised, finely minced with scissors, and suspended in 0·25 M aqueous sucrose solution buffered to pH 7·2 with 0·02 M Tris HCl. Ten per cent (w/v) homogenates were prepared by homogenizing tissues for 30 sec at 600 rev/min in a Thomas tissue grinder fitted with a motor-driven teflon pestle. Homogenates were centrifuged for 1 h at 105,000 *g*, and the supernatant fraction (cytosol) was used for enzyme assays. The extracts were kept at 0–4°C during and after preparation.

*Adenosine deaminase assay.*—The assay method was based on that of Kalckar (1947). The sample cuvette contained 2·35 ml of 0·15 M potassium-phosphate buffer, pH 7·2, and 0·05 ml of enzyme preparation. Reference cuvettes contained 2·45 ml of the buffer and 0·05 ml of enzyme. After equilibration to 37°C, reaction was started by addition to the sample cuvette of 0·1 ml of 2·5 mM adenosine solution. The reaction was followed at 265 nm in a Cary model 118 CX double-beam spectrophotometer, fitted with an automatic multiple sample programmer and cuvette positioner. Under these conditions reaction rates were linear for at least 5 min, and proportionate to amount of enzyme up to at least 6× the usual amount. The molar optical-density change at 265 nm for the ADA reaction was redetermined for the present study, at physiological conditions of pH and ionic strength, and found to be $8{\cdot}5 \times 10^3$/cm. ADA rates measured in this way were about 72% of $V_{max}$, for both liver and hepatoma enzymes. Higher rates could be obtained by using increased adenosine concentrations, but this resulted in greater interference from stray light, with consequent increase in optical noise and poorer reproducibility. Thus, ADA activity was determined as described above, and $V_{max}$ values were calculated by multiplying measured rates by 1·4.

*Adenosine kinase assay.*—This was adapted from the method of DeJong and Kalkman (1973). The reaction mixture contained Tris-HCl buffer (7·5 μmol) dithioerythritol (0·03 μmol), KCl (0·15 μmol), $MgCl_2$ (0·075 μmol), ATP (0·15 μmol), phosphoenolpyruvate (0·15 μmol), $^3H$-labelled adenosine (0·015 μmol; 0·25 μCi), pyruvate kinase (0·75 iu) and enzyme preparation (usually 10 μl of a 5-fold dilution of 105,000 *g* supernatant fraction of 10% homogenate) in a final reaction volume of 0·15 ml. The various components were added to microfuge tubes, mixed by a 10 sec centrifugation and then incubated at 37°C for 10 min. Reaction blanks used boiled enzyme. Reaction was stopped by boiling for 3 min, and precipitated protein was sedimented by a 30 sec spin in the microfuge. Aliquots of the supernatant fraction (25 μl) were applied to 2·5 cm-diameter discs of anion-exchange paper (Whatman, DE81). Unreacted adenosine was removed by 3 successive 5 min washes in 150 ml of aqueous ammonium formate (1 mM) followed by 5 min washes in distilled water and absolute ethanol. The washed discs were then combusted in a Packard Model 306 sample oxidizer; combustion products were absorbed in Monophase 40 scintillation fluid (Packard) and measured by scintillation counting. Under these conditions, reaction rate was proportional to time up to 4 min, and to enzyme amount up to 25 μl of 2% homogenate.

*Cell culture.*—Hepatoma cultures were maintained in McCoy's medium 5A, supplemented with penicillin (100 u/ml) and streptomycin (100/μg/ml), and 10% horse serum (which, unlike foetal calf serum, contains no ADA). The origins, properties, and maintenance of the Morris Hepatoma Culture Lines 3924A, 8999R and 8999S are described by Jackson, Williams and Weber (1976) and Jackson and Weber (1976). Novikoff cells were the subline NISI-67 (Plagemann and Swim, 1966). Hepatoma 7795 was used as the subline MHlCl (Richardson, Tashjian and Levine, 1969). Under these conditions mean log-phase doubling times were: Novikoff, 12 h; 3924A, 16 h; 8999R, 31 h; 8999S, 63 h; 7795, 95 h.

*Hepatocyte preparation.*—This was by the method of Berry and Friend (1969), modified as described by Harris (1975).

*Assays of ATP, ADP and AMP.*—These were measured chromatographically on a 10×0·6 cm column of Aminex A-27 anion exchange resin (Bio-Rad Laboratories, Richmond, California) eluted with alkaline citrate buffer gradients, as described by Khym (1975).

*Protein assay.*—Protein was determined by the method of Lowry *et al.* (1951), using bovine plasma albumin as standard.

### RESULTS

### *Kinetic properties of liver and hepatoma enzymes*

For kinetic studies, ADA and AK were partially purified from rat liver and from rapidly growing Hepatoma 3924A as described by Streeter *et al.* (1974). The 105,000 *g* supernatant fractions from 30 g of tissue were treated with solid ammonium sulphate, and the protein precipitating between 55% and 90% saturation was re-dissolved in 3·5 ml of a buffer consisting of 10 mM Tris HCl (pH 7·5), 10 mM $MgCl_2$, 1 mM dithiothreitol and 0·075 M KCl (Buffer A) and applied to a column of Sephadex G-100 (2 cm$^2$ × 90 cm) eluted with the same buffer. Peaks of AK and ADA overlapped. The active fractions were pooled and applied to a 7 cm$^2$ × 20 cm column of DEAE-cellulose (Whatman DE52). This column was eluted with a linear gradient for which the starting buffer was Buffer A without KCl, and the high-ionic-strength eluent was the same buffer containing 0·3 M KCl. As described by Streeter *et al.* (1974), AK and ADA were completely separated by this column. The ADA, about 45-fold purified relative to the 105,000 *g* supernatant fraction, was used for kinetic studies without further purification. The AK was concentrated by precipitating with ammonium sulphate, re-dissolved, and further purified on a column (4 cm$^2$ × 60 cm) of Sephadex G-75 eluted with Buffer A. The resulting AK preparation, about 50-fold purified relative to the 105,000 *g* supernatant fraction, was used for kinetic studies.

Michaelis constants for adenosine of the ADA preparations was measured by assaying the enzyme in triplicate at a range of adenosine concentrations (5, 10, 20, 35, 50, 75, 100, 150 and 250 $\mu$mol/l). Saturation curves were roughly hyperbolic, with no indication of high substrate inhibition. The Michaelis constants, as calculated by the method of Wilkinson (1961) were $37 \pm 4$ $\mu$molar (liver ADA) and $45 \pm 7$ $\mu$molar (Hepatoma 3924A enzyme). Both liver and hepatoma ADA were subject to product inhibition by inosine; results for the rat liver enzyme are shown in double reciprocal form in Fig. 1. The inhibition was competitive with adenosine, and $K_{i,slope}$, as calculated by the computer programme "COMP" (Cleland, 1963) was 280 $\mu$M; the corresponding value for the hepatoma 3924A enzyme was 345 $\mu$M.

AK activity was compared at a range of adenosine concentrations (0·25, 0·5, 1·0, 1·5, 2·0, 5·0, 10·0 and 25·0 $\mu$M). No excess-substrate inhibition was seen at the higher adenosine concentrations. Michaelis constants of AK for adenosine, as calculated by the method of Wilkinson (1961), were 1·3 $\mu$M (liver) and 1·5 $\mu$M (Hepatoma 3924A). In view of the observation of Murray (1968) that free ATP, rather than Mg-ATP, was the best phosphate donor for AK, ATP saturation curves were studied at a constant $Mg^{2+}$ concentration of 0·5 mM which was found to be the

FIG. 1.—Inhibition of adenosine deaminase (ADA) from rat liver by inosine. A 45-fold purified enzyme preparation was used, and assays were done as described in the text. Open circles, control; closed circles, inosine at 38 $\mu$M; open squares, inosine at 72 $\mu$M.

FIG. 2.—Effect of ATP concentration on the velocity of the AK reaction. A 50-fold purified rat liver enzyme preparation was used, and standard assay conditions, except that phosphoenolpyruvate and pyruvate kinase were omitted from the reaction mixture. A: Saturation curve. B: The same data, at ATP concentrations of 2 M and above, displayed as a Hill plot, where a = measured velocity divided by $V_{max}$.

optimal level in the present study when measured at an ATP concentration of 1 mM. The ATP saturation curve for the liver enzyme is shown in Fig. 2(a). The apparent $K_m$ value, calculated from the rates obtained at ATP concentrations of 1 mM or less, was 230 $\mu$M. At ATP concentrations of 2 mM and above, as shown in Fig. 2(a), strong product inhibition was caused by ATP. Fig. 2(b) is a Hill plot of the same data, for ATP concentrations of 2 mM and above. The slope of this plot is $-2{\cdot}0$. Since AK is not believed to be a multiple subunit enzyme, a possible explanation of the high value of the Hill coefficient for excess ATP inhibition is that ATP binds to both the AMP and ADP product sites. When the experiment was repeated with Mg-ATP instead of free ATP, very similar results were obtained, with 50% inhibition reached at an Mg-ATP concentration of 3·4 mM. The AK from Hepatoma 3924A was also susceptible to excess-substrate inhibition by ATP. A concentration of 3·5 mM (free ATP) gave 50% inhibition, relative to the rate obtained at 1 mM, which was the optimal ATP concentration for the tumour enzyme, as it was in normal liver AK. ADP and AMP were effective inhibitors of rat liver

TABLE I.—*Inhibition of Rat Liver Adenosine Kinase (AK) by Ribonucleotides**

| Nucleotide | Concn. (mM) | AK activity (% of uninhibited control) |
|---|---|---|
| ADP | 0·13 | 65 |
| | 0·33 | 45 |
| | 1·00 | 4 |
| AMP | 0·13 | 83 |
| | 0·33 | 63 |
| | 1·00 | 47 |
| GDP | 0·33 | 86 |
| | 1·00 | 65 |
| GTP | 2·00 | 70 |
| UTP | 2·00 | 95 |
| UDP | 0·33 | 97 |
| | 1·00 | 96 |
| UMP | 0·33 | 102 |
| | 1·00 | 93 |
| | 2·00 | 85 |
| CDP | 0·33 | 103 |

* A 50-fold purified preparation of enzyme was used for these experiments; activity was measured by the standard assay, as described in "Materials and Methods".

AK; lesser degrees of inhibition were caused by other ribonucleotides (Table I).

*Organ and tissue distribution of ADA and AK*

The activities of the two enzymes were measured in a series of organs and tissues of adult Wistar rats. Results are summarized in Table II. In the AK assays, dipyridamole (0·8 mM) was added to inhibit ADA, since in some tissues the ADA activity greatly exceeded that of AK. Preliminary experiments with the liver enzymes showed that this concentration of dipyridamole inhibited ADA by 97% and AK by 13%. The highest specific activity of AK was found in liver. Many tissues, however, have higher ADA activity than liver, and the highest activities were present in intestinal mucosa and thymus. Table II also shows the ratio of maximal activities for the 2 enzymes. Only in liver and erythrocytes was AK activity higher than ADA. The 2 activities were equal in brain, and in all the other tissues examined ADA activity was higher than AK activity.

*Activities of ADA and AK in tumours*

Specific activities of the 2 enzymes, as measured by the standard assays, for 12 transplantable rat hepatomas, are summarized in Fig. 3. In these studies, the mean ADA specific activity in cytosol preparations from 18 control rats was 1760±70 (s.e. mean) nmol/h/mg cytosol protein. Specific activity of ADA in cytosol preparations from the hepatomas is shown as a percentage of the control liver value. In the hepatomas of slow or medium growth rate, with intervals be-

TABLE II.—*Adenosine Deaminase (ADA) and AK Activities in Rat Tissues**

| Organ or tissue | Cytosol protein (mg/g wet wt) | ADA | AK | ADA/AK |
|---|---|---|---|---|
| Liver | 93±3 | 1,510 (100) | 4,270 (100) | 0·35 |
| Kidney cortex | 74±4 | 6,410 (424) | 1,520 (36) | 4·2 |
| Heart | 58±4 | 3,870 (256) | 1,280 (30) | 11·0 |
| Testis | 47±9 | 3,880 (257) | 1,160 (27) | 3·3 |
| Spleen | 71±6 | 13,900 (923) | 1,010 (24) | 13·8 |
| Lung | 79±5 | 10,900 (719) | 987 (23) | 11·0 |
| Epididymal fat pad | 18±3 | 2,270 (150) | 733 (17) | 3·1 |
| Brain (cerebra) | 32±2 | 713 (47) | 713 (17) | 1·0 |
| Skeletal muscle (gastrocnemius) | 55±4 | 1,130 (75) | 687 (16) | 1·7 |
| Small intestine (mucosa) | 58±6 | 41,900 (2,780) | 610 (14) | 68·7 |
| Thymus | 53±2 | 47,700 (3,160) | 419 (10) | 113·9 |
| Bone marrow (femoral) | 84±10 | 1,800 (119) | 207 (4·8) | 8·7 |
| Erythrocytes | 360±32 | 36 (2·4) | 144 (3·3) | 0·25 |
| Peripheral leucocytes | 90±6 | 627 (42) | 60 (1·4) | 10·4 |
| Blood plasma | 63±3 | 21 (1·4) | 0 | — |

* Measured as described in the Methods section; in the AK assay, 0·8 mM dipyridamole was present, to inhibit ADA. Activities expressed as nmol/h/mg protein (in parentheses, as % of the liver activities). Protein was measured by the method of Lowry *et al.* (1951), except for erythrocyte haemolysates, where the biuret method was used (Gornall *et al.*, 1949). Figures are mean values for quadruplicate samples.

FIG. 3.—Activities of ADA and AK in cytosol preparations from rat hepatomas. The activities are expressed as % of levels in control rat livers. The absolute activities of the 2 enzymes in the control livers are given in the text. Also shown is the ratio of $V_{max}$ of the 2 enzymes, and the cytosol protein content of the hepatomas, expressed as a % of the mean control liver value (91 mg/g wet wt).

tween successive transplants ranging from 12 to 5 months, ADA activity was in the normal range (80% to 130% of liver activity). However, in 5 hepatoma lines of rapid growth rate (interval between transplants of 1 month or less) ADA activity was significantly increased, at least doubled in every case, and reaching 4·2-fold in the most rapidly growing liver tumour.

The mean±s.e. mean for AK activity in normal liver from 19 control rats was 3840±160 nmol/h/mg cytosol protein. Fig. 3 shows hepatoma activity as percentages of the control liver value. The AK activity was decreased in all the tumours; this decrease was statistically significant ($P<0·05$ in Student's $t$ test) in every case except Hepatoma 20. In the slow and medium growth-rate hepatomas specific activity was 64–85% of the control liver value, but in tumours of rapid growth rate the AK activity was further decreased (37% of control, or less) falling to 6% of control in the most rapidly growing hepatoma. Thus the AK activity in hepatomas showed a negative correlation with growth rate. Kendall's rank-correlation coefficient for AK activity and growth rate was −0·697, statistically a highly significant correlation ($P<0·01$). Fig. 3 also presents the ratio of maximal activities of ADA/AK in the hepatomas. In the various normal liver samples examined, this ratio was in the range 0·35–0·46. The ratio was slightly higher in the slow and medium growth-rate neoplasms (0·47–0·76) and considerably increased in the hepatomas of rapid growth rate. In the fastest-growing hepatoma this ratio reached 32·1, a higher value than in any of the normal tissues examined, except intestinal epithelium and thymus.

Activities of the two enzymes were also measured in two transplantable kidney tumours. In 8 samples of normal adult rat renal cortex, specific activity of ADA was 6·15 μmol/h/mg protein (soluble protein 76 mg/g wet wt). In kidney tumour MK1, specific activity of ADA was 23% of that in the kidney control; in kidney tumour MK3, ADA specific activity was 22% of control (protein contents were: MK1, 78mg/g; MK3, 72 mg/g). The tumour ADA activities given are means of quadruplicate samples, with scatter between replicates not greater than ±12%. Specific activity of AK in normal kidney cortex was 1·54 μmol/h/mg protein (mean for 8 samples). Activity in kidney tumour MK1 was 80% of the normal kidney control, and ADA in kidney tumour MK3 was 53% of control (means of quadruplicate samples). Ratios of ADA/AK in this study were thus: control renal cortex, 4·0; kidney tumour MK1, 1·15; kidney tumour MK3, 1·66.

### *ADA and AK in regenerating liver*

Fig. 4 shows activity of ADA and AK at a number of intervals after partial hepatectomy or sham laparotomy. The opera-

FIG. 4.—Activities of ADA and AK in rat liver after partial hepatectomy. All points are mean values for groups of 5 or 6 animals. Vertical bars indicate s.e. mean. Experimental details are given in the text. Symbols: ○, AK in sham-operated rat liver; □, AK in regenerating rat liver; ●, ADA in sham-operated rat liver; ■, ADA in regenerating rat liver.

tions were timed such that all animals were killed at 10.00 a.m. Cytosol fractions were prepared and ADA and AK activities measured by the standard assays. At the 6 intervals examined between 12 and 96 h after operation, the activity of ADA showed no significant change. In contrast, the AK activity of regenerating liver was already significantly decreased, relative to sham-operated controls, by 12 h after the operations. At 24 h the AK activity in the regenerating livers was about 60% of the sham-operated control value; it remained at this level at 48 h. By 72 h after operation the AK activity in the regenerating liver samples had increased to near the normal range. This decrease in AK activity in the regenerating liver samples, in the presence of unaltered ADA activity, resulted in a ratio of ADA/AK of 0·70 in the 24 h regenerating liver, compared to a ratio of 0·41 in unoperated control samples of ACI/N rat liver.

### *ADA and AK in differentiating liver and kidney*

Activities of the 2 enzymes in differentiating liver samples, ranging from 17-day foetal rats to mature 60-day-old rats are shown in Table III. Specific activity of ADA in the liver of foetal and neonatal rats was significantly higher than in adult rat liver, gradually declining with increasing age. Specific activity of AK in the liver of 17-day embryos was significantly lower than the adult level. However, it reached the adult value by the 20th day of gestation, and thereafter remained in the adult range until maturity. Because the cells of foetal or neonatal liver are much smaller than mature hepatocytes (Table III) the number of cells/g of liver is much higher in the immature animals. Thus, if activity of ADA and AK were expressed per cell (Table III), both enzymes, on this basis, had lower activity in the foetal or neonatal liver than in adult liver. It was interesting to compare the ratio ADA/AK at the various stages of development, since this ratio is independent of the units of the individual activities; the ADA/AK ratio in the 17-day foetal liver was 1·5 (3·7× the adult liver ratio), declining with increasing age, and approaching the adult value by 31 days after birth.

Table III also shows ADA and AK activities in neonatal kidney. Specific activity of ADA was higher, and that of AK lower, in neonatal kidney than in adults. Activities of both enzymes were lower in the neonatal kidney, on a per-cell basis, than in adult kidney cortex. As with the liver, the ratio of ADA/AK was higher in neonatal kidney than in the mature organ.

### *Growth inhibition of cultured hepatoma cells by adenosine*

Fig. 5 summarizes the growth-inhibitory effect of adenosine against 5 rat hepatoma lines in culture. It was shown by Ishii and Green (1973) that the growth-inhibitory effect of adenosine is cell-density depend-

TABLE III.—*ADA and AK in Developing Liver and Kidney of Wistar Rats**

| Tissue and age of animal | Cell count ($10^6$/g wet wt) | Cytosol protein (mg/g wet wt) | ADA per g wet wt | ADA µmol/h per mg protein | ADA per $10^9$ cells | AK per g wet wt | AK micromoles/h per mg protein | AK per $10^9$ cells | ADA/AK |
|---|---|---|---|---|---|---|---|---|---|
| Liver: | | | | | | | | | |
| 17-day foetal | 720 | 46 | 136 (96)† | 2·96 (201) | 189 (29) | 91 (27) | 1·98 (56) | 126 (8) | 1·50 |
| 20-day foetal | 660 | 54 | 133 (94) | 2·46 (168) | 202 (31) | 192 (56) | 3·56 (100) | 291 (19) | 0·69 |
| 1-day postnatal | 590 | 68 | 138 (98) | 2·03 (138) | 234 (36) | 247 (72) | 3·63 (102) | 419 (27) | 0·56 |
| 6-day postnatal | 530 | 77 | 155 (110) | 2·01 (137) | 292 (46) | 308 (90) | 3·99 (112) | 581 (37) | 0·50 |
| 22-day postnatal | 360 | 83 | 162 (115) | 1·95 (133) | 450 (70) | 372 (109) | 4·48 (126) | 1033 (66) | 0·44 |
| 31-day postnatal | 320 | 86 | 152 (108) | 1·77 (120) | 476 (74) | 357 (104) | 4·15 (117) | 1116 (72) | 0·43 |
| 42-day postnatal | 280 | 90 | 148 (105) | 1·65 (112) | 529 (82) | 333 (97) | 3·70 (104) | 1189 (77) | 0·45 |
| 60-day postnatal | 220 | 96 | 141 (100) | 1·47 (100) | 640 (100) | 342 (100) | 3·56 (100) | 1550 (100) | 0·41 |
| Kidney: | | | | | | | | | |
| 1-day postnatal | 630 | 46 | 402 (92) | 8·74 (154) | 638 (54) | 50 (37) | 1·08 (63) | 79 (22) | 8·08 |
| 5-day postnatal | 595 | 50 | 507 (116) | 10·14 (179) | 852 (72) | 79 (59) | 1·57 (92) | 132 (37) | 6·44 |
| 60-day postnatal | 370 | 77 | 437 (100) | 5·67 (100) | 1180 (100) | 132 (100) | 1·72 (100) | 358 (100) | 3·30 |

* Measured as described in the Methods section. Data are mean values for quadruplicate samples.

† In parentheses as % of appropriate control value (adult liver or kidney).

FIG. 5.—Effect of adenosine on growth of established hepatoma lines in culture. Cultures were initiated at $2 \times 10^5$ cells in 10 ml medium in 25 $cm^2$ flasks. Adenosine was added to the indicated concentration, and further equal additions of adenosine were made after 24 and 48 h. Cultures were counted after 72 h, and results were expressed as increase in cell count relative to the increase in cell count of control cultures.

ent, so all cultures were initiated at the same density (20,000/ml). Preliminary studies showed that more than 90% of adenosine was removed from the medium of these cultures after 24 h, so successive additions of adenosine were made at the time of initiation and after 24 and 48 h. This removal of adenosine was presumably caused by the intracellular ADA, since the horse serum used to supplement the culture medium contained no ADA. Cultures were counted after 72 h, and the cell counts, as percentages of control values (no adenosine) are plotted in log-log form in Fig. 5 as a function of adenosine concentration. The sensitivity of the cells to adenosine toxicity showed a negative correlation with growth rate (correlation coefficient −0·83). Table IV shows the 50% growth-inhibitory concentrations ($ID_{50}$) for adenosine, as measured in 72 h experiments with the 5 hepatoma lines in culture. Specific activities of ADA and AK in the 5 lines are also tabulated (with the enzyme activities of isolated normal liver cells for comparison). In these experiments, cells were sedimented at 1100 *g* for 7 min, suspended in Tris-HCl buffer, pH 7·2, 0·05 M, and then successively frozen in liquid $N_2$ and thawed in a 37°C water bath, 4 times, to lyse the cells. Lysates were centrifuged at 105,000 *g* for 60 min, and activities measured by the standard assays in the supernatant fraction. ADA and AK activities in the cultured hepatoma cells were in general similar to the levels in the solid tumours. All cell lines showed lower AK activity than normal liver; the decreases were small in the slow-growing lines 7795 and 8999S, and more marked in the faster-growing tumours. ADA activity was higher in all the hepatoma lines than in normal liver. Its activity was 133% of liver activity in the slowest-growing line, 7795, and 2–3·5-fold the liver value in the other hepatoma lines. The ratio ADA/AK had a highly significant correlation with growth rate ($r=0·979$) and with the adenosine $ID_{50}$ value ($r=0·921$). It thus appears that, for growth in the presence of extraneous adenosine, the increased ability of hepatomas to deaminate adenosine and decreased capacity to phosphorylate adenosine confers a selective advantage.

*Incorporation of adenosine into ribonucleotide pools*

Lund *et al.* (1975) showed that incubation of isolated rat hepatocytes with adenosine produced large increases in cellular ATP, and smaller increases in ADP and AMP. We designed similar experiments using Hepatoma 8999R and Novikoff cell lines, to determine the extent to which this response to adenosine is modified by the changes in activity of the competing enzymes ADA and AK, as observed in hepatomas. Isolated normal hepatocytes

TABLE IV.—*ADA and AK Activities (as μmol/h/mg protein) in Cultured Hepatoma Cells*†

| Cell type | Doubling time (h) | $ID_{50}$ for adenosine (mM) | ADA | AK | ADA/AK |
|---|---|---|---|---|---|
| Liver | —* | — | 1·50 (100)‡ | 3·56 (100) | 0·42 |
| 7795 | 95 | 0·04 | 1·99 (133) | 3·08 (87) | 0·65 |
| 8999S | 63 | 0·04 | 3·97 (265) | 3·21 (90) | 1·24 |
| 8999R | 31 | 0·06 | 3·14 (209) | 1·64 (46) | 1·92 |
| 3924A | 16 | 0·21 | 4·30 (287) | 0·92 (26) | 4·65 |
| Novikoff | 12 | 1·4 | 5·31 (354) | 0·65 (18) | 8·12 |

* Normal liver cells do not divide in culture under these conditions.
† Activities are means of duplicate assays on triplicate cultures.
‡ As % of normal liver value.

and cultured 8999R and Novikoff hepatoma cells were incubated in McCoy's Medium 5A (~$3\times10^7$ cells/5 ml) supplemented with 10% horse serum, and containing various concentrations of $^3H$-labelled adenosine (40 mCi/mmol). After 60 min at 37°C, cells were extracted with 4·5% perchloric acid, and extracts were neutralized with 4 N KOH, and analysed for ATP, ADP and AMP by the chromatographic method of Khym (1975). The radioactivity in each of these peaks was measured by scintillation counting. Loss of adenosine from the incubation medium was measured optically, as described by Moellering and Bergmeyer (1974). Data are shown in Table V. Results with hepatocytes at the highest adenosine concentration studied (300 μM) were similar to the results obtained by Lund *et al.* (1975) with 500 μM adenosine: total adenine nucleotides increased by more than 100%, most of the increase being ATP. Twenty per cent of the adenosine utilized was recovered as adenine nucleotides, the remainder presumably being deaminated. At the lower adenosine concentrations studied (10 and 30 μM) more than half the adenosine was recovered in adenine nucleotides. In cells of Hepatoma 8999R, 60 min incubation with 300 μM adenosine gave a 40% increase in adenine nucleotides. At this adenosine level, 90% was deaminated, and the proportion of adenosine deaminated at the lower concentrations studied was higher than in liver. In Novikoff cells the effect of extracellular adenosine on adenine nucleotide levels was even smaller, and most of the adenosine was deaminated at all 3 concentrations studied. Although it is possible that some of the radioactive adenine nucleotides were formed by the

TABLE V.—*Effects of Extracellular Adenosine on Liver and Hepatoma Cells*

| Cell type | Adenosine conc. (μM) | Adenine nucleotide contents (μmol/$10^9$ cells)* | | | | % adenosine deaminated |
|---|---|---|---|---|---|---|
| | | ATP | ADP | AMP | Total | |
| Liver | 0 | 9·6 | 2·9 | 1·2 | 13·7 (100)† | — |
| | 10 | 9·3 | 3·2 | 1·0 | 13·5 (99) | 26 |
| | 30 | 11·0 | 3·0 | 1·2 | 15·2 (111) | 44 |
| | 300 | 21·4 | 4·5 | 1·7 | 27·6 (201) | 80 |
| 8999R | 0 | 5·9 | 1·8 | 0·8 | 8·5 (100) | — |
| | 10 | 6·0 | 1·9 | 0·9 | 8·8 (104) | 55 |
| | 30 | 6·4 | 2·0 | 0·9 | 9·3 (109) | 75 |
| | 300 | 8·8 | 2·1 | 0·9 | 11·8 (139) | 90 |
| Novikoff | 0 | 5·0 | 1·5 | 0·6 | 7·1 (100) | — |
| | 10 | 5·1 | 1·7 | 0·8 | 7·6 (107) | 77 |
| | 30 | 5·4 | 1·7 | 0·7 | 7·8 (110) | 87 |
| | 300 | 6·6 | 1·6 | 0·7 | 8·9 (125) | 93 |

* Means of analyses of duplicate cultures. Incubation time was 60 min.
† In parentheses, as % of the control value (without adenosine).

route adenosine→inosine→hypoxanthine →IMP→adenylosuccinate→AMP, the rate-limiting enzyme of that sequence (adenylosuccinate synthetase) is less active than AK, by a factor of between 7 and 100 in the various cell types (Jackson, Morris and Weber, 1977) and the proportion of adenosine label recovered in adenine nucleotides showed a good correlation with the ADA/AK ratio in the 3 cell types.

### DISCUSSION

The examination of kinetic properties of ADA and AK suggests that the metabolic fate of adenosine is controlled by at least 3 factors. The first is the ratio of activities of these 2 competing enzymes. The elucidation of the behaviour of the activities of these enzymes in normal and neoplastic tissues was the primary aim of the present study, and the conclusions will be discussed in detail below. The second controlling factor is the ratio of $K_m$ values for the 2 enzymes. The very low $K_m$ for adenosine of AK (1·3 $\mu$M), compared to the higher value of ADA (37 $\mu$M), ensures that adenosine is conserved at low concentrations. Data in Table V show that the proportion of adenosine deaminated increased with concentration. This arrangement may enable the cell to recycle small amounts of adenosine resulting from cellular AMP phosphatase activity, whilst protecting it from the toxic effects of high extracellular adenosine production. The third factor that controls adenosine utilization is the feedback inhibition of AK by AMP, ADP and ATP. Data shown in Table I and Fig. 2 indicate that these effects are appreciable at physiological nucleotide levels, though these inhibitions do not provide a completely effective homoeostatic mechanism, since incubation of cells with high concentrations of adenosine led to an elevated cellular adenine-nucleotide content. The product inhibition of ADA by inosine was feeble, and may be considered as of no physiological importance.

The results of the ADA tissue and organ distribution study (Table II) closely resembled earlier surveys of this enzyme in rat tissues (Clarke *et al.*, 1952; Purzycka, 1962; Brady and O'Donovan, 1965). The novel aspect of the present distribution study was the simultaneous determination of ADA and AK, which made possible accurate information concerning the ratio of these competing enzymes. Noteworthy observations were: both ADA activity and the ADA/AK ratio were highest in thymus, and next highest in intestinal epithelium. AK activity was highest in liver, and next highest in renal cortex. In brain the ADA/AK ratio was about unity; this ratio was $<1$ in erythrocytes and liver, and $>1$ in all other tissues. The ADA/AK ratio was lowest in erythrocytes; this would presumably result in a proportionately high incorporation of adenosine into adenine nucleotides, consistent with this route being an important source of erythrocyte ATP, as proposed by Lerner and Lowy (1974). The ADA activity and ADA/AK ratio were considerably higher in cardiac muscle than in skeletal muscle, which was of interest in view of the cardiotoxicity of adenosine.

In general, ADA activity and the ADA/AK ratio were high in organs of cell proliferation (*e.g.* thymus, intestinal epithelium). In the hepatoma series, a clear pattern was evident. AK activity was lower than the normal liver value in all 12 hepatomas. In the tumours of slow or intermediate growth rate, with intervals between successive transplant generations of 5 months or more, the AK activity was in the range of 64–85% of the liver value. In 5 rapidly growing hepatomas (transplant intervals 1 month or less) there were more marked decreases in the AK activity. These rapidly growing tumours were the only ones to show significant changes in ADA activity, relative to normal liver (at least doubled). Thus it is clear that the consequence of these enzyme activity changes in hepatomas would be that proportionately more adenosine would be deaminated, and less phosphorylated, than in normal liver, with the most marked effects observed in hepatomas of rapid growth rate. This prediction from the enzyme levels is supported by the data of Table V, in which adenosine phosphorylation and deamination were measured in suspensions of isolated normal hepatocytes and cultured hepatoma cells. The increased deamination and decreased phosphorylation of adenosine appear to confer increased resistance to adenosine toxicity upon the cells. The data of Table IV show that Novikoff rat hepatoma cells, with an ADA/AK ratio 12× that of Hepatoma 7795, are 35× as resistant to the growth-inhibitory effect of adenosine.

The changes in activity of ADA and AK cannot be regarded as stringently linked with the neoplastic transformation. Activity of ADA showed no significant change in the slow-growing hepatomas (Fig. 3) and low values in peripheral leucocytes from chronic lymphocytic leukaemia (Scholar and Calabresi, 1973; Meier, Coleman and Hutton, 1976). However, the marked increases of ADA activity in rapidly growing hepatomas (Fig. 3) and in acute leukaemia cells (Smyth and Harrap, 1975; Meier *et al.*, 1976) suggest that this enzyme may correlate with the process of malignant progression. A lack of correlation of ADA activity with cell proliferation *per se* seems to be indicated by the unchanged values in regenerating liver and neonatal liver. On the other hand, AK, which exhibited a negative correlation with growth rate in the hepatomas, also showed a marked and statistically significant decrease in regenerating liver, and was low in foetal and neonatal liver. The decrease in AK activity is thus found in both malignant and nonmalignant states of cell proliferation, though it was most marked in the rapidly-growing hepatomas. The changes which we observed in ADA and AK activities appear to be quantitative; no differences in kinetic or chromatographic properties were seen when the two enzymes were partially purified from rat liver and Hepatoma 3924A. The question whether multiple forms of these enzymes occur in rat liver and hepatomas will form the basis of future studies.

The reciprocal behaviour of the opposing enzymes ADA and AK, most markedly expressed in the rapidly growing neoplasms, is in line with previous observations on the behaviour of antagonistic enzymes in carbohydrate, purine and pyrimidine metabolism (Weber, 1977). The changes reported above in activity of the competing enzymes ADA and AK should confer a selective advantage upon hepatoma cells in the presence of extracellular adenosine. That ADA activity influences adenosine toxicity was demonstrated by Harrap and Paine (1977), who showed that coformycin, a potent and specific inhibitor of ADA, greatly increased the sensitivity of lymphoblasts to adenosine. In solid tumours, which often contain areas of necrotic tissue, the adenosine concentration might be appreciable, and the protection from adenosine toxicity afforded by increased deamination and reduced phosphorylation could have a marked effect on tumour survival. To establish the magnitude of adenosine toxicity *in vivo*, direct measurement of tissue adenosine levels will be necessary.

This work was supported by research grants from the United States Public Health Service (CA 13526, CA 05034, and CA 10792). The authors are grateful to Mr L. Soliven for skilled technical assistance.

### REFERENCES

Berry, M. N. & Friend, D. S. (1969) High-yield Preparation of Isolated Rat Liver Parenchymal Cells. *J. Cell. Biol.*, **43**, 506.

Brady, T. G. & O'Donovan, C. I. (1965) A Study of the Tissue Distribution of Adenosine Deaminase in Six Mammal Species. *Comp. Biochem. Physiol.*, **14**, 101.

Chan, T. S., Ishii, K., Long, C. & Green, H. (1973) Purine Excretion by Mammalian Cells Deficient in Adenosine Kinase. *J. Cell Physiol.*, **81**, 315.

Clarke, D. A., Davoll, J., Phillips, F. S. & Brown, G. B. (1952) Enzymic Deamination and Vasodepressor Effects of Adenosine Analogues. *J. Pharmac. exp. Ther.*, **106**, 219.

Cleland, W. W. (1963) Computer Programmes for Processing Enzyme Kinetic Data. *Nature*, **198**, 463.

DeJong, J. W. & Kalkman, C. (1973) Myocardial Adenosine Kinase: Activity and Localization Determined with Rapid, Radiometric Assay. *Biochim. biophys. Acta*, **320**, 388.

DeLamirande, G., Allard, C. & Cantero, A. (1958) Purine-metabolizing Enzymes in Normal Rat Liver and Novikoff Hepatoma. *Cancer Res.*, **18**, 952.

Dissing, J. & Knudsen, B. (1972) Adenosine Deaminase Deficiency and Combined Immunodeficiency Syndrome. *Lancet*, ii, 1316.

Giblett, E. R., Anderson, J. E., Cohen, F., Pollara, B. & Meuwissen, H. J. (1972) Adenosine Deaminase Deficiency in Two Patients with Severely Impaired Cellular Immunity. *Lancet*, ii, 1067.

Gornall, A. G., Bardawill, C. J. & David, M. M. (1949) Determination of Serum Proteins by Means of the Biuret Reaction. *J. biol. Chem.*, **177**, 751.

Harrap, K. R. & Paine, R. M. (1977) Adenosine Metabolism in Cultured Lymphoid Cells. *Adv. Enzyme Regul.*, **15**, 169.

Harris, R. A. (1975) Studies on the Inhibition of Hepatic Lipogenesis by $N^6$, $O^{2'}$-Dibutyryl Adenosine 3′, 5′-Monophosphate. *Archs. biochem. Biophys.*, **169**, 168.

Higgins, G. M. & Anderson, R. M. (1931) Experimental Pathology of the Liver. I. Restoration of the Liver of the White Rat Following Partial Surgical Removal. *Archs. Pathol.*, **12**, 186.

Ishii, K. & Green, H. (1973) Lethality of Adenosine for Cultured Mammalian Cells by Interference with Pyrimidine Biosynthesis. *J. Cell Sci.*, **13**, 429.

Jackson, R. C., Morris, H. P. & Weber, G. (1977) Enzymes of the Purine Ribonucleotide Cycle in Rat Hepatomas and Kidney Tumors. *Cancer Res.*, **37**, 3057.

Jackson, R. C. & Weber, G. (1976) Enzyme Pattern Directed Chemotherapy. The Effects of Combination of Methotrexate, 5-Fluorodeoxyuridine and Thymidine on Rat Hepatoma Cells *in vitro*. *Biochem. Pharmacol.*, **25**, 2613.

Jackson, R. C., Williams, J. C. & Weber, G. (1976) Enzyme Pattern Directed Chemotherapy: Synergistic Interaction of 3-Deazauridine with D-Galactosamine. *Cancer Treatment Rep.*, **60**, 835.

Kalckar, H. M. (1947) Differential Spectrophotometry of Purine Compounds by Means of Specific Enzymes. *J. biol. Chem.*, **167**, 461.

Khym, J. X. (1975) An Analytical System for Rapid Separation of Tissue Nucleotides at Low Pressures on Conventional Anion Exchangers. *Clin. Chem.*, **21**, 1245.

Lerner, M. H. & Lowy, B. A. (1974) The Formation of Adenosine in Rabbit Liver and Its Possible Role as a Direct Precursor of Erythrocyte Adenine Nucleotides. *J. biol. Chem.*, **249**, 959.

Lowry, O. H., Rosebrough, N. J., Farr, A. L. & Randall, R. J. (1951) Protein Measurement with the Folin Phenol Reagent. *J. biol. Chem.*, **193**, 265.

Lund, P., Cornell, N. W. & Krebs, H. A. (1975) Effect of Adenosine on the Adenine Nucleotide Content and Metabolism of Hepatocytes. *Biochem. J.*, **152**, 593.

Meier, J., Coleman, M. S. & Hutton, J. J. (1976) Adenosine Deaminase Activity in Peripheral Blood Cells of Patients with Haematological Malignancies, *Br. J. Cancer*, **33**, 312.

Moellering, H. & Bergmeyer, M. U. (1974) Adenosine. In *Methods of Enzymatic Analysis 2nd edn.* Ed. M. U. Bergmeyer. Weinheim: Verlag Chemie.

Murray, A. W. (1968) Some Properties of Adenosine Kinase from Ehrlich Ascites Tumour Cells. *Biochem. J.*, **106**, 549.

Parkman, R., Gelfand, E. W., Rosen, F. S., Anderson, A. & Hirschhorn, R. (1975) Severe Combined Immunodeficiency and Adenosine Deaminase Deficiency. *New Engl. J. Med.*, **292**, 714.

Plagemann, P. G. W. & Swim, H. E. (1966) Replication of Mengovirus. I. Effect on Synthesis of Macromolecules by Host Cell. *J. Bacteriol.*, **91**, 2317.

Purzycka, J. (1962) AMP and Adenosine Aminohydrolases in Rat Tissues. *Acta Biochim. Polon.*, **9**, 83.

Reid, E. & Lewin, I. (1957) Adenosine Deaminase, Nucleoside Phosphorylase and Xanthine Oxidase in Liver Tumours. *Br. J. Cancer*, **11**, 494.

Richardson, U. I., Tashjian, A. H. & Levine, L. (1969) Establishment of a Clonal Strain of Hepatoma Cells which Secrete Albumin. *J. cell. Biol.*, **40**, 236.

Scholar, E. M. & Calabresi, P. (1973) Identification of the Enzymatic Pathways of Nucleotide Metabolism in Human Lymphocytes and Leukaemic Cells. *Cancer Res.*, **33**, 94.

Smyth, J. F. & Harrap, K. R. (1975) Adenosine Deaminase Activity in Leukaemia. *Br. J. Cancer*, **31**, 544.

Streeter, D. G., Simon, L. N., Robins, R. K. & Miller, J. P. (1974) The Phosphorylation of Ribavirin by Deoxyadenosine Kinase from Rat Liver. Differentiation between Adenosine and Deoxyadenosine Kinase. *Biochemistry*, **13**, 4543.

Weber, G. (1977) Enzymology of Cancer Cells. *New Engl. J. Med.*, **296**, 486, 541.

Wilkinson, G. N. (1961) Statistical Estimations in Enzyme Kinetics. *Biochem. J.*, **80**, 324.